# A treatment with a boiled aqueous extract of *Hancornia speciosa* Gomes leaves improves the metabolic status of streptozotocin-induced diabetic rats

**DOI:** 10.1186/s12906-020-02919-2

**Published:** 2020-04-17

**Authors:** Leila S. Neto, Rafaianne Q. Moraes-Souza, Thaigra S. Soares, Marcelo S. Pinheiro, Thaís Leal-Silva, Juliana C. Hoffmann, Madileine F. Américo, Kleber E. Campos, Débora C. Damasceno, Gustavo T. Volpato

**Affiliations:** 1 Laboratory of System Physiology and Reproductive Toxicology, Institute of Biological and Health Sciences, Federal University of Mato Grosso (UFMT), Av. Valdon Varjão 6390, Barra do Garças, Mato Grosso State 78600-000 Brazil; 2 Laboratory of Experimental Research on Gynecology and Obstetrics, Botucatu Medical School, Univ Estadual Paulista_Unesp, Distrito de Rubião Júnior s/n, Botucatu, São Paulo State 18618-687 Brazil

**Keywords:** *Hancornia speciosa*, Diabetes, Medicinal plants, Biochemical, Rats

## Abstract

**Abstract:**

**Background:**

*Hancornia speciosa* is usually used in Brazilian folk medicine to treat diabetes. The hypothesis of the present study is that this medicinal plant exerts beneficial effects on hyperglycemia, preventing diabetic complications. Therefore, the aim of this study was to evaluate the treatment effect of the aqueous extract of *H. speciosa* leaves on metabolic parameters of diabetic rats.

**Methods:**

The *H. speciosa* extract (400 mg/Kg) was administered to both nondiabetic and severely diabetic female Wistar rats by gavage. The Oral Glucose Tolerance Test was performed and the area under the curve (AUC) was estimated on day 17 of pregnancy. After 21 days of treatment, the animals were anesthetized and killed to obtain organ weights. Blood samples were collected for an analysis of serum biochemical parameters.

**Results:**

After treatment with the *H. speciosa* extract, the parameters of nondiabetic rats remained unchanged. In treated diabetic rats, glycemia, AUC, dyslipidemia parameters, and relative organ weights were decreased compared with nontreated diabetic rats. Severely diabetic rats showed decompensated hyperglycemia, polydipsia, hyperphagia and dyslipidemia. However, the aqueous extract of *H. speciosa* leaves decreased diabetes complications (indicating a lack of toxicity), reduced blood glucose levels, and exerced lipid-lowering effects.

**Conclusion:**

Based on or findings, the *H. speciosa* leaf extract may be a safe and promising candidate treatment for diabetes and other diseases.

## Background

Medicinal plants have positively affected human health. These plants are consumed mostly based on personal experience or traditional knowledge [1]. In the last two decades, the use of alternative and complementary medicine has considerably increased worldwide [2] because herbal medicine is considered an innate part of the culture that has been used and disseminated over the generations [3].

One plant widely used in folk medicine is *Hancornia speciosa* Gomes (family Apocynaceae), known as mangaba. Mangaba is a native Brazilian tree, and Gomes described the fruitful species in 1812. This tree thrives in a dry tropical and subtropical climate, and it has been found in several regions of Brazil. *H. speciosa* is tolerant to periods of drought and higher temperatures, and presents a good vegetative development [4]. Previous phytochemical studies identified different classes of *H. speciosa* leaf constituents, including cyclitols, cinnamic acids, flavonoids, steroids, triterpenes and phenolic compounds [4–6].

In folk medicine, this plant is used as a treatment for several diseases such tuberculosis, lower back pain, hypertension, dysmenorrhea, respiratory and venereal diseases, dermatosis and ulcers, obesity, and *Diabetes mellitus* [7, 8]. *H. speciosa* exerts vasodilatory [9, 10], cancer chemopreventive [5, 11], and antihypertensive effects [7, 12], and stimulates the migration and/or proliferation of fibroblasts [13]. From a scientific perspective, a specific study designed to investigate the plant safety and efficacy is necessary by developing simple bioassays for biological standardization, pharmacological and toxicological evaluation, and by developing animal models [14]. Only one study has evaluated the hypoglycemic effect of *H. speciosa* leaves. According to Pereira et al. [15], a single dose of the *H. speciosa* leaves (300 mg/kg of ethanolic extract) reduced blood glucose level in male Swiss mice, inhibiting intestinal α-glucosidase activity in vitro and stimulating glucose uptake in adipocytes. However, this study was not performed on an animal model of diabetes. Thus, the hypothesis of the present study is that this medicinal plant exerts beneficial effects on hyperglycemia, preventing diabetic complications. Therefore, the aim of this study was evaluate the treatment effect of the aqueous extract of *H. speciosa* leaves on metabolic parameters of diabetic rats.

## Methods

### Extraction of plant materials

The *Hancornia speciosa* leaves were collected from Pontal do Araguaia, Mato Grosso State, Brazil (15°91′51″ S and 52°27′62″ W), between April and May 2014 in the morning. The plant was identified and authenticated by Prof. Maryland Sanchez Lacerda Pedroni from the Botanical Department of Mato Grosso Federal University (UFMT), where a voucher specimen (UFMT 02285) has been deposited. The plant leaves were dried at 50 °C for a 24 h period in an aerated stove, ground and a powder was prepared, similar to the method used to prepare the folk medicine. The *H. speciosa* aqueous extract was prepared by boiling one liter (L) of water containing 60 g (g) of the powdered plant leaves for 5 min (min). The extract was agitated and covered until it reached room temperature. The residue was removed by filtration (1 mm pore size filter) and the extract was then suitably concentrated in a rotary evaporator. A sample was separated to determination the concentration of the solid component, and the extract was divided into aliquots stored at − 20 °C until further use.

### Animals

Female Wistar rats (190–210 g) were obtained from the UFMT Vivarium and were maintained under standard laboratory conditions (22 ± 3 °C, 12-h light/dark cycle), with pelleted food (Purina® rat chow, São Paulo, Brazil) and tap water provided ad libitum. The procedures and animal handling protocols were authorized by the Ethics Committee for Animal Research of the UFMT, Brazil (Protocol number 23108.001991/13–1).

After 2 weeks of acclimation, diabetes was induced by injecting streptozotocin (STZ, Sigma Chemical Company®, St. Louis, MO) into fasting rats. STZ was intravenously (i.v.) injected at a dose of 40 mg/kg after it was dissolved in citrate buffer (0.1 M, pH 6.5). Non-diabetic (control) rats received and i.v. injection of citrate buffer. Blood glucose concentrations were measured by conventional glucometer 7 days after diabetes induction; glucose concentrations exceeding 300 mg/dL confirmed the diabetic state, and these rats were included in the diabetic group [16].

### Experimental groups and treatment

After confirming the successful establishment of the diabetes model, the rats were divided into four experimental groups (*n* = 11 animals/group): Control - rats treated with vehicle (water); Treated Control - rats treated with the *H. speciosa* extract; Diabetic - diabetic rats treated with vehicle, and Treated Diabetic - diabetic rats treated with the *H. speciosa* extract. The *H. speciosa* extract was administered at a dose of 400 mg/kg/day by oral gavage for 21 days. Body weight, water intake, food intake and blood glucose levels were also evaluated weekly in the morning. The animals were not fasted.

### Oral glucose tolerance test

The oral glucose tolerance test (OGTT) was performed on day 17 of treatment to evaluate the development of inadequate glucose metabolism. OGTT is regularly used to determine a clinical diagnosis of diabetes. After an overnight fast of 8 h, blood was collected from the tail of the rats by venipuncture to determine the glycemic status (time point 0). Afterwards, the treated groups received the plant extract and the control group received vehicle according to the method described by Chayarop et al. [17]. After 30 min, a glucose solution (200 g/L) was prepared at a final dose of 2 g/kg body weight and was intragastrically administered to rats. Next, blood glucose concentrations were measured at 30, 60 and 120 min [18, 19]. Glucose concentrations in blood collected from the tail vein were measured using a conventional glucometer. Glucose responses during the OGTT were evaluated by estimating the total area under the curve (AUC) using the trapezoidal method [20].

### Data collection

After 21 days of treatment, the non-fasted rats were anesthetized with an injection of sodium pentobarbital (Thiopentax® - 120 mg/kg) and blood samples were collected after decapitation for and analysis of biochemical biomarkers. Moreover, the heart, liver, spleen, and kidneys were removed and weighed. The relative weight was determined by calculating the ratio of the weight of each organ (grams) to the body weight on day 21 of pregnancy minus the gravid uterus weight (grams) × 100. The result was reported in grams/100 g body weight.

The blood samples were collected in anticoagulant-free test tubes, incubated on in ice for 30 min and then centrifuged at 1300×*g* for 10 min at 4 °C. The supernatant was collected as the serum fraction and stored at − 80 °C until a further analysis of the biochemical parameters. Serum concentrations of total protein were determined using a colorimetric method with the Biuret reagent [21]. The total cholesterol, triglyceride, and high-density lipoprotein (HDL-c) concentrations, as well as alanine aminotransferase (ALT), and aspartate aminotransferase (AST) activities were estimated using an enzymatic method [22] with Winner® assay kits. The values are reported in milligrams per deciliter (mg/dL). The non-HDL cholesterol concentration (Non-HDL-c) was calculated as follows: non-HDL cholesterol = total cholesterol - HDL cholesterol [23].

### Statistical analysis

One-way analysis of variance (ANOVA) followed the Student-Newman-Keuls test was used to compare the mean values of all parameters. Differences were considered statistically significant when *p* < 0.05.

## Results

### Glucose parameters

**Fig. 1:**
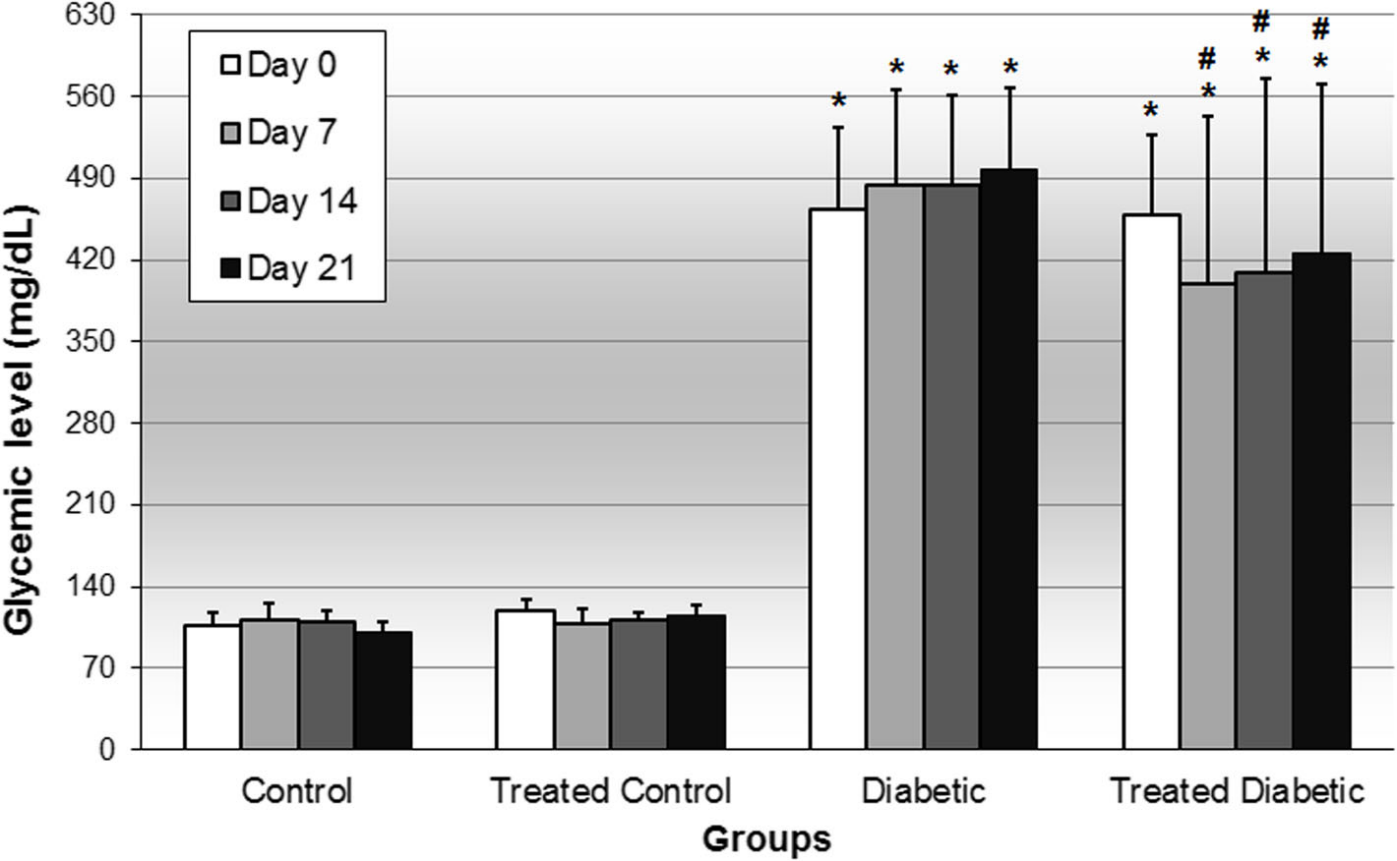
Glycemic level on days 0, 7, 14 and 21 of control and diabetic rats treated or not with a *Hancornia speciosa* aqueous extract (400 mg/kg) during 21 days. *Data are shown as mean ± standard deviation.*^*a*^*p < 0.05 compared with the control group (ANOVA followed Student-Newman-Keuls test).*^*b*^*p < 0.05 compared with the diabetic group (ANOVA followed Student-Newman-Keuls test)*

**Fig. 2:**
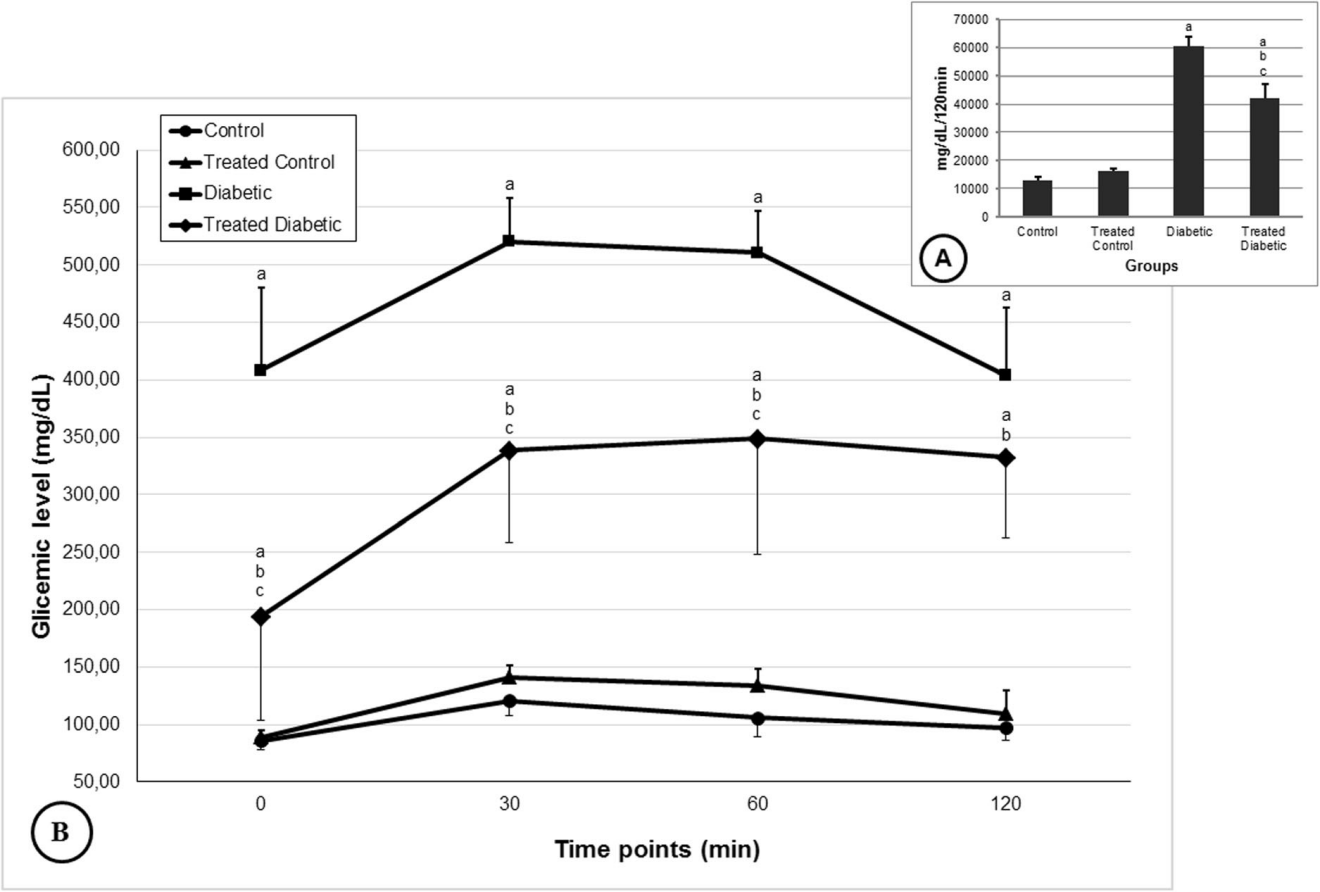
Oral glucose tolerance test (OGTT) and area under the curve (AUC) at day 17 of treatment of control and diabetic rats treated or not with a *Hancornia speciosa* aqueous extract (400 mg/kg) during 21 days. *Data are shown as mean ± standard deviation.*^*a*^*p < 0.05 compared with the control group (ANOVA followed Student-Newman-Keuls test)*^*b*^*p < 0.05 compared with treated control group (ANOVA followed Student-Newman-Keuls test)*^*c*^*p < 0.05 compared with the diabetic group (ANOVA followed Student-Newman-Keuls test)*

Glycaemia was approximately 110 mg/dL in control groups. In diabetic groups, glucose levels were greater than 400 mg/dL. The *H. speciosa* aqueous extract treatment did not significantly alter hyperglycemia compared to the control group, but it decreased the glycemic level compared to the diabetic group (Fig. 1). In the OGTT test, the glycemic level was not different between the non-diabetic treated group and the control group at all time points analyzed. The diabetic non-treated animals presented increased glycemic levels at all time points compared to the control groups. The diabetic animals treated with the plant extract presented hyperglycemia at all time points of the OGTT compared to both non-diabetic animals and exhibited decreased hyperglycemia at 0, 30 and 60 min compared to the diabetic group. The control groups showed an area under the curve (AUC) approximately 13,000 mg/dL/120 min. The rats from the diabetic group showed a higher AUC compared than control groups, reaching approximately 60,000 mg/dL/120 min. The *H. speciosa* extract treatment did not change the AUC compared with the control group, but it reduced the AUC compared to the untreated diabetic group (Fig. 2).

### Indirect toxicity analyses

No differences in body weight were observed among the experimental groups. The diabetic groups displayed significantly higher water intake and food consumption than the control groups. In addition, the plant treatment reduced food consumption by the diabetic animals compared to untreated diabetic animals on days 14 and 20 of the experiment (Table 1).

**Table 1:** Body weight, water intake and food consumption of control and diabetic rats treated or not with *Hancornia speciosa* aqueous extract (400 mg/kg) during 21 days

	***Groups***			
	*Control*	*Treated Control*	*Diabetic*	*Treated Diabetic*
***Body weight (g)***				
Day 0	218.2 ± 30.3	210.6 ± 24.7	222.6 ± 37.3	211.1 ± 16.8
Day 7	226.4 ± 30.0	198.8 ± 20.9	217.6 ± 30.3	214.2 ± 23.1
Day 14	229.0 ± 28.8	211.4 ± 23.4	217.0 ± 29.0	204.1 ± 32.4
Day 20	230.8 ± 28.3	214.2 ± 23.4	219.8 ± 30.6	211.1 ± 31.3
***Water intake (mL)***				
Day 0	35.7 ± 8.0	24.8 ± 2.9	87.1 ± 28.0 ^a^	79.4 ± 13.8 ^a^
Day 7	33.7 ± 5.4	29.6 ± 6.1	90.9 ± 29.0 ^a^	81.2 ± 17.7 ^a^
Day 14	36.2 ± 5.1	36.7 ± 4.4	106.0 ± 33.4 ^a^	90.6 ± 25.8 ^a^
Day 20	33.9 ± 4.5	33.2 ± 5.7	112.4 ± 34.6 ^a^	102.9 ± 20.0 ^a^
***Food consumption (g)***				
Day 0	16.5 ± 3.1	13.3 ± 3.6	22.3 ± 7.1 ^a^	22.3 ± 5.4 ^a^
Day 7	16.2 ± 2.5	13.6 ± 3.4	26.3 ± 7.9 ^a^	23.1 ± 4.9 ^a^
Day 14	16.3 ± 2.2	17.5 ± 3.6	31.1 ± 7.5 ^a^	25.0 ± 4.8 ^a b^
Day 20	16.4 ± 2.0	15.5 ± 1.8	33.6 ± 9.3 ^a^	23.8 ± 7.4 ^a b^

*Data are shown as mean ± standard deviation (SD)*

^*a*^*p < 0.05 compared with the control group (ANOVA followed Student-Newman-Keuls test)*

^*b*^*p < 0.05 compared with the diabetic group (ANOVA followed Student-Newman-Keuls test)*

The diabetic group showed increased weights of all organs evaluated in this study compared with the control group. The treated diabetic group presented a reduction in the relative organ weights compared to the diabetic group, except for the relative kidney weight (Table 2).

**Table 2:** Relative organ weight of control and diabetic rats treated or not with *Hancornia speciosa* aqueous extract (400 mg/kg) during 21 days

	Groups			
	*Control*	*Treated Control*	*Diabetic*	*Treated Diabetic*
Heart (g/100 g)	0.33 ± 0.02	0.39 ± 0.05	0.50 ± 0.07 ^a^	0.35 ± 0.02 ^a b^
Liver (g/100 g)	3.22 ± 0.29	3.36 ± 0.23	4.87 ± 0.37 ^a^	3.61 ± 0.40 ^a b^
Spleen (g/100 g)	0.22 ± 0.04	0.22 ± 0.01	0.29 ± 0.03 ^a^	0.24 ± 0.02 ^a b^
Right kidney (g/100 g)	0.32 ± 0.04	0.36 ± 0.03	0.54 ± 0.04 ^a^	0.44 ± 0.04 ^a b^
Left kidney (g/100 g)	0.33 ± 0.05	0.33 ± 0.02	0.54 ± 0.04 ^a^	0.41 ± 0.04 ^a b^

*Data are shown as mean ± standard deviation (SD)*

^*a*^*p < 0.05 compared with the control group (ANOVA followed by Student-Newman-Keuls test)*

^*b*^*p < 0.05 compared with the diabetic group (ANOVA followed by Student-Newman-Keuls test)*

### Biochemical parameters

The diabetic group showed higher serum triglyceride, total cholesterol, and non-HDL-c levels, as well as higher ALT and AST activities than the control group. Treatment with the *H. speciosa* reduced the total cholesterol, triglyceride, and non-HDL-c levels, and ALT and AST activities in the diabetic group compared with the untreated diabetes condition (Table 3).

**Table 3:** Biochemical parameters of control and diabetic rats treated or not with *Hancornia speciosa* aqueous extract (400 mg/kg) during 21 days

	***Groups***			
	*Control*	*Treated Control*	*Diabetic*	*Treated Diabetic*
Total protein (g/dL)	4.4 ± 0.3	4.5 ± 0.3	4.8 ± 0.2	4.3 ± 0.2
Tryglicerides (mg/dL)	90.0 ± 23.6	82.4 ± 43.0	606.7 ± 334.1 ^a^	455.2 ± 113.9 ^a c^
Cholesterol (mg/dL)	80.0 ± 4.6	75.2 ± 18.2	114.2 ± 19.2 ^a^	66.1 ± 9.2 ^b^
HDL-c (mg/dL)	58.9 ± 6.8	49.4 ± 7.9	62.4 ± 9.6	58.3 ± 28.6
Non-HDL-c (mg/dL)	22.3 ± 8.8	35.8 ± 10.7	52.4 ± 21.5 ^a^	29.6 ± 5.7 ^b^
ALT (U/l)	79.0 ± 8.8	63.2 ± 21.9	171.4 ± 94.7 ^a^	91.3 ± 28.6 ^b^
AST (U/l)	171.6 ± 17.3	136.2 ± 21.6	375.4 ± 193.8 ^a^	159.5 ± 37.8 ^b^

*Data are shown as mean ± standard deviation (SD)*

^*a*^*p < 0.05 compared with the control group (ANOVA followed by Student-Newman-Keuls test)*

^*c*^*p < 0.05 compared with treated control group (ANOVA followed by Student-Newman-Keuls test)*

^*b*^*p < 0.05 compared with the diabetic group (ANOVA followed by Student-Newman-Keuls test)*

## Discussion

Several herbal medicines are used to treat diabetes. However, some of the reported benefits have not been confirmed in laboratory experiments. Experimental models of diabetes in rats have been widely used by researchers to assess the actual hypoglycemic effect of the plants [24–26]. In this study, the diabetic groups presented hyperglycemia, indicating that streptozotocin effectively induces diabetes, consistent with the findings of other studies [25, 27–30]. The OGTT is used in the clinic to diagnose diabetes and investigate antidiabetic agents by measuring the change in plasma glucose levels in response to oral glucose administration [31].

In the present study, the *Hancornia speciosa* treatment (400 mg/kg) was administred to determine the plasma glucose-lowering effect. In normal animals, the treatment did not alter the plasma glucose levels or the area under the curve (AUC) during the OGTT. Nevertheless, the hyperglycemia and AUC were reduced in the rats with uncontrolled diabetes, confirming the effectiveness of the extract at reducing the blood glucose level. However, the plant did not reduce hyperglycemia to normal levels probably because of the severe diabetes status, which is characterized by severe hyperglycemia (glucose level greater than 300 mg/kg) [32]. Pereira et al. [15] verified that the ethanolic *H. speciosa* extract (300 mg/kg) exerted a potential anti-diabetic effect related to the inhibition of intestinal α-glucosidase activity and stimulation of glucose uptake by adipocytes. These authors proposed that this effect might be mediated by the terpenoids, steroids and tannins present in the ethanolic *H. speciosa* extract.

The body weight loss represents a common effect of diabetes. Despite the increased appetite, insulin deficit reduces all anabolic processes and increases catabolic processes, contributing to further body weight loss [33]. This biological condition was not observed in the present study, in contrast to previous studies using this diabetic model [32, 34]. Perhaps the glycemic level, although high, was not sufficient to decrease the weight of these animals during the experimental period. Both diabetic groups showed polydipsia and hyperphagia, which were interpreted as evidence of the hyperglycemic status. These symptoms are a compensatory mechanism to deprive cells of glucose and to increase the glucose concentration in the urine [33]. However, in the present study, the treatment with plant extract decreased the food consumption on days 14 and 21 of treatment, which was probably related to the decrease in the glycemic levels of these animals. The treatment with 400 mg/kg of the *H speciosa* extract did not alter the food and water consumption of the non-diabetic animals, indicating that this plant does not exert an indirect toxic effect on healthy animals. The aqueous extract of *H. speciosa* leaves also showed no toxicity, as evidenced by the absence of a relevant clinical sign and the absence of deaths throughout the experimental period. The relative weights of the organs and biochemical profile also represent toxicity signals [35]. In our study, the diabetic rats presented a higher relative heart weight. Hyperglycemia is a hyperosmotic state that contributes to increasing the myocardial water content, leading to heart dysfunction [36] and affecting the heart weight. In our study, the weight of the kidney (hypertrophy) was increased in diabetic groups, which might be due to local changes in the production of one or more growth factors and/or their receptors [37], glucose over-utilization and subsequent increases in glycogen synthesis, lipogenesis and protein synthesis [38]. However, treatment with the plant extract reversed the diabetic-induced damage to these organs (heart and kidneys), which was associated with decreased hyperglycemia, and consequently, reduce the relative weights of these organs.

Our findings also showed an increased relative weight of the liver in the diabetic group that as directly induced by hyperglycemia. Changes in triglyceride output by hepatocytes in diabetic animals may lead to fat accumulation in the liver, the induction of hepatic lipogenesis and an increase in intrahepatic fat synthesis [39], which might contribute to the abnormal liver weight. Diabetes-induced alterations in the liver structure lead to changes in the ALT and AST enzyme activities [40], as verified in our study. The literature confirms that hepatic enzymatic analyses have been used as signs of tissue injury and toxicity [41]. The *H. speciosa* treatment reduced ALT and AST activities, indicating the hepatoprotective effect of the plant, which also might contribute to decreasing the liver weight in these rats. Moreover, the *H. speciosa* extract decreased cholesterol levels to approximately normal values. The reduced blood cholesterol concentration might be related to the actions of some phenolic compounds present in this plant, which might inhibit 3-hydroxy-3-methylglutaryl-CoA (HMG-CoA) reductase, a rate-limiting enzyme in cholesterol biosynthesis. Similarly, other studies also suggested hypocholesterolemic effect of phenolic compound after the administration of a plant extract that was mediated by the inhibition of HMG-CoA reductase [42, 43]. *H. speciosa* also contains triterpenes, such as lupeol and α- and β-amirin [15], which potentially reduce the cholesterol levels of diabetic rats. In addition, the *H. speciosa* extract decreased triglyceride and non-HDL-c levels (represented by the lipoproteins VLDL, IDL and LDL) [23] in diabetic rats. The decrease in hyperglycemia observed after treatment with the plant extract might contribute to the mechanism regulating the hepatic gluconeogenesis and the lipid profile in the diabetic animals, decreasing glycerol and fatty acid levels, and consequently triglyceride and non-HDL-c levels, as observed in the present study.

However, limitations of this study were the utilization of only one dose during the experiment and the lack of a positive drug control, which might be included in a subsequent investigation to confirm the specific antidiabetic effect of the *H. speciosa* extract. In addition, this *H. speciosa* extract should be tested in another diabetes model presenting a lower glycemic intensity, such as less intense hyperglycemia, reproducing the level of hyperglycemia in humans with type 2 *Diabetes mellitus* [44].

## Conclusion

In conclusion, severe diabetes is characterized by uncontrolled hyperglycemia, polydipsia, hyperphagia and dyslipidemia in rats. The aqueous extract of *H. speciosa* leaves treatment at the tested dose shows no signs of toxicity, reduces blood glucose levels and exerts lipid-lowering effects. Thus, based on the findings of this investigation, the extract of *H. speciosa* potentially represents a safe and intersting candidate treatment for diabetes and other diseases.

## Data Availability

The datasets used and/or analyzed during the current study are available from the corresponding author on reasonable request.

## Abbreviations

ALT: Alanine aminotransferase
ANOVA: Analysis of variance
AST: Aspartate aminotransferase
AUC: Area under the curve
CNPq: Conselho Nacional de Desenvolvimento Científico e Tecnológico
g: gram
*H. speciosa*: *Hancornia speciosa*
HDL-c: High-density lipoprotein
HMG-CoA: 3-hydroxy-3-methylglutaryl-CoA
i.v: intravenous
L: Litter
LDL: Low-density lipoprotein
mg/dL: milligrams per deciliter
mg/kg: milligrams per kilogram
mL: milliliter
Non-HDL-c: Non-HDL-cholesterol
OGTT: Oral glucose tolerance test
SD: Standard deviation
STZ: Streptozotocin
UFMT: Federal University of Mato Grosso
VLDL: Very-low-density lipoprotein
